# Effects of Postpartal Relative Body Weight Change on Production Performance, Serum Biomarkers, and Fecal Microbiota in Multiparous Holstein Cows

**DOI:** 10.3390/ani15091252

**Published:** 2025-04-29

**Authors:** Siyuan Zhang, Yiming Xu, Tianyu Chen, Duo Gao, Jingjun Wang, Yimin Zhuang, Wen Jiang, Guobin Hou, Shuai Liu, Shengli Li, Wei Shao, Zhijun Cao

**Affiliations:** 1 College of Animal Science, Xinjiang Agricultural University, Urumqi 830052, China; zsy19930910@163.com (S.Z.); xuyiming1028@163.com (Y.X.); 17881135032@163.com (W.J.); dksw@xjau.edu.cn (W.S.); 2 State Key Laboratory of Animal Nutrition and Feeding, International Calf and Heifer Organization, College of Animal Science and Technology, China Agricultural University, Beijing 100193, China; chentianyu@cau.edu.cn (T.C.); godo0910@163.com (D.G.); wangjingjun@cau.edu.cn (J.W.); z1164323345@163.com (Y.Z.); houguobin1996@163.com (G.H.); liushuaicau@cau.edu.cn (S.L.);

**Keywords:** NEB, body weight change, Holstein, postpartum, fecal microbiota

## Abstract

This study aimed to determine effects of postpartal relative body weight change (PRBWC) on production performance, serum biomarkers, and the relation between PRBWC and gastrointestinal microbiota. A total of 59 multiparous cows participated in this research. Every cow’s PRBWC was calculated by the following equation: PRBWC = (BW_21_ − BW_0_)/BW_0_ × 100%, in which BW_21_ refers to body weight on Day 21 post-calving and BW_0_ refers to body weight on the day of parturition. Among the 59 enrolled cows, cows with the top 21 ranked PRBWC values were categorized into the high PRBWC (H-PRBWC) group; cows with the bottom 21 ranked PRBWC values were categorized into the low PRBWC (L-PRBWC) group. PRBWC did not have significant influences on average daily milk yield (ADMY). However, on Day 21, cows in the H-PRBWC group displayed significantly higher body weight (BW) and body condition scores (BCS) (BW, *p* = 0.02; BCS, *p* < 0.01). Additionally, levels of serum glucose (GLU) and albumin (ALB) were significantly higher in the H-PRBWC group on Day 21 (GLU, *p* = 0.05; ALB, *p* < 0.01), while the lipopolysaccharide-binding protein (LBP) level was significant lower (*p* = 0.03). Moreover, the microbiota of fecal samples on Day 0 (FE0) differed notably between groups, as evidenced by various alpha diversity indices, including Shannon (*p* = 0.02), Simpson (*p* = 0.03), Pielou_e *(p* = 0.02), and principal coordinate analysis (*p* = 0.002). The relative abundances of *Monoglobus*, *norank_f__UCG-010*, and *Christensenellaceae_R-7_group* were significantly higher in the H-PRBWC group (*p* < 0.05), while the relative abundances of *Clostridium_sensu_stricto_1*, *Turicibacter*, and *Romboutsia* were significantly lower (*p* < 0.05). Pathways related to amino acid biosynthesis were significantly enriched in the FE0 of the H-PRBWC group, while pathways involved in carbohydrate metabolism were significantly upregulated in the FE0 of the L-PRBWC group. This study argues the potential of PRBWC to describe alteration of energy status in the postpartum, evidenced by production performance, serum biomarkers, and the fecal microbiota.

## Simple Summary

To assess the depletion of a cow’s energy reserves during the postpartum, considering precision and individual variability, we introduced a new metric known as postpartal relative body weight change (PRBWC). Certain production and serum parameters associated with an individual’s energy levels supported the effectiveness of PRBWC in evaluating relative energy deficits throughout the postpartum. Furthermore, fecal microbiota on Day 0 appeared to have an effect on PRBWC: the fecal microbiota on Day 0 demonstrated intergroup discrepancies between cows with high PRBWC or low PRBWC.

## 1. Introduction

One of the crucial phases in the milk production cycle is the peripartum stage, where dairy cows encounter various challenges, including energy deficits, altered hormone profiles, metabolic disorders, and compromised immune function [1]. Among these challenges, negative energy balance (NEB) has become a primary focus of research in dairy science during the transition period. Severe NEB often manifests in the postpartum, resulting in an unsuccessful transition period of dairy cows that negatively impacts the cow’s health, subsequent milk production, and fertility [2]. Consequently, identifying a parameter to select cows with better energy status under the same feeding management may prove beneficial.

The calculation of energy balance (EB) is a direct method for assessing the level of energy deficit. Still, the process can be quite complex, requiring measurements of multiple parameters, including heat production, feed intake, dry matter, nutrient, and enzyme-soluble organic substance contents of the diet; body weight (BW); milk yield; and milk composition [3]. Although serum biomarkers, such as ketone bodies, can accurately reflect the energy status [4], invasive blood collection is not practical for routine use in production settings. In commercial farms, body weight change (BWC) and body condition score (BCS) can serve as non-invasive alternatives. BWC represents the change in body mass and provides an absolute estimate toward energy deficit. However, it may not accurately reflect an individual animal’s energy status, as the individual variations of BW on Day 0 were neglected: the same amount of postpartal BWC may result in different levels of influence on cows with different values of BW on Day 0. On the other hand, although BCS offers a relative measure that indicates the degree of energy status in a specific cow at the end of the postpartum, the unit is divided into 0.25 increments, relying solely on visual estimation, which limits its precision [5]. Furthermore, excessive energy intake may preferentially increase the visceral depots rather than subcutaneous depots of adipose tissues [6], which cannot be easily observed by visual measurement. Therefore, a relative parameter with a reasonable degree of precision is necessary to describe changes in energy reserves while considering individual differences in postpartum. In this study, we introduce a parameter called postpartal relative body weight change (PRBWC) to address these needs.

Some serum biomarkers involved in energy metabolism, oxidative status, hepatic function, and inflammatory response alter during the postpartum according to the degree of energy deficit [1,7], which may indirectly reflect the cow’s energy status to a certain extent. Cows are heterotrophs that depend on feed to fulfill all their energy requirements, and the digestion of feed is closely linked to the profile and functions of the rumen microbiota involved in fermentation [8]. Furthermore, previous studies have shown that the gut microbiota can regulate metabolism in hosts that are affected by energy-related metabolic diseases, such as obesity and diabetes [9,10]. Consequently, this study aimed to investigate whether PRBWC has the potential to effectively assess the level of a cow’s energy deficit during the postpartum on the basis of production performance parameters and serum biomarkers related to the cow’s energy status. It also explored the relationships between PRBWC and fecal microbiota.

## 2. Materials and Methods

### 2.1. Ethics Statement

The animal care protocol was approved by the Animal Care and Use Committee of China Agricultural University (Protocol Number: AW42202202-4-1).

### 2.2. Animals and Experimental Design

Animal experiments were conducted at the Tianmu Farm in Gansu Province, China. Fifty-nine multiparous cows were enrolled in this study, starting from parturition (Day 0) and continuing until 21 days post-calving (Day 21). From this 59-cow herd, 42 cows were selected and categorized into two groups based on their PRBWC values (Days 0 to 21). The 21 cows with the highest PRBWC formed the H-PRBWC group, while the 21 cows with the lowest values were assigned to the L-PRBWC group. PRBWC was calculated as follows:

PRBWC = (BW_21_ ^1^ − BW_0_ ^2^)/BW_0_ × 100%

^1^ BW on Day 21; ^2^ BW on Day 0.

### 2.3. Diets and Feeding Management

All 59 cows received formulated rations throughout the postpartum. The composition and nutrient levels of the diet are demonstrated in Table S1. Cows were provided with a total mixed ration three times daily at 07:00, 14:00, and 21:00, and they always had free access to feed and clean water, except during milking.

### 2.4. Sampling and Measurement

A total of 42 cows were assigned to either the H-PRBWC group or the L-PRBWC group on the basis of the rank of the PRBWC value among the 59 cows. However, measurements and sampling were conducted on all 59 cows. Both body weight on Day 0 (BW_0_) and body condition score on Day 0 (BCS_0_) were measured immediately after parturition; both body weight on Day 21 (BW_21_) and body condition score on Day 21 (BCS_21_) were measured after milking and before the morning feeding on Day 21. The cows were scored on a scale of 1 to 5, with increments of 0.25 units, as illustrated in the Elanco BCS chart [11]. The cows were milked three times per day, and the daily milk yield was recorded from Day 0 to Day 147 using a rotary milking machine (DeLaval, Co., Ltd., Tianjin, China). The average daily milk yield (ADMY) was calculated as follows:

ADMY ^1^ = sum of daily milk yield during period/days of period

^1^ ADMY, average daily milk yield.

On Day 21, all experimental cows underwent fasting tail vein blood collection by a 10 mL vacuum tube without any anticoagulant within 1 h prior to the morning feeding. Then the tubes with blood samples were placed on ice for 2 h before being centrifuged at 3500× *g* for 20 min at 4 °C. After centrifugation, the supernatants were collected in sterile 1.5 mL frozen storage tubes for serum samples, which were then stored at −20 °C until analysis.

The serum biomarkers were classified into 4 categories regarding their roles in the following physiological activities: energy metabolism, including β-hydroxybutyric acid (BHBA), blood urea nitrogen (BUN), glucose (GLU), insulin-like growth factor-1 (IGF-1), non-esterified fatty acids (NEFA), and total cholesterol (TC); hepatic function, including albumin (ALB), haptoglobin (HPT), total bilirubin (TBIL), and total protein (TP); oxidative status, malondialdehyde (MDA), superoxide dismutase (SOD), and total antioxidant capacity (T-AOC); and inflammatory response, interleukin (IL)-1β, IL-6, lipopolysaccharide-binding protein (LBP), serum amyloid A (SAA), and tumor necrosis factor (TNF)-α. The information of the instruments or commercial kits used for serum biomarker measurement and the corresponding manufacturers are listed in Table S2.

Ruminal fluid and fecal samples from all experimental cows were collected on both Day 0 and Day 21. For the ruminal fluid collection, approximately 50 mL of ruminal fluid was obtained by intubation using esophageal tubes and syringes, then filtered through four layers of sterile cheesecloth and stored in 10 mL sterile tubes. Fecal samples were collected directly from the rectum using sterile gloves and stored in 5 mL sterile tubes. Both the ruminal and fecal samples were subsequently stored at −80 °C for further taxonomic analysis via 16S rRNA sequencing.

### 2.5. DNA Extraction, PCR Amplification, and Sequencing

The bacterial genomic DNA was extracted from rumen fluid samples or fecal samples on Day 0 and Day 21 from the H-PRBWC group (*n* = 21) and the L-PRBWC group (*n* = 21) with the E.Z.N.A.^®^ Soil DNA Kit (Omega Bio-Tek, Norcross, GA, USA) according to the manufacturer’s instructions. The quality and concentration of extracted DNA were determined by 1.0% agarose gel electrophoresis and a Nano-Drop2000 spectrophotometer (Thermo Scientific, Waltham, MA, USA), and then DNA samples were kept at −80 °C until further analysis. The hypervariable region V3–V4 of the bacterial 16S rRNA gene was amplified with the primer pairs 338F (5′-ACTCCTACGGGAGGCAGCAG-3′) and 806R (5′-GGACTACHVGGGTWTCTAAT-3′) by a T100 Thermal Cycler PCR thermocycler (BIO-RAD, Hercules, CA, USA) according to the instructions. The PCR product was extracted from 2% agarose gel and purified using the PCR Clean-Up Kit (YuHua, Shanghai, China) according to the manufacturer’s instructions and quantified using Qubit 4.0 (Thermo Fisher Scientific, Waltham, MA, USA). Purified amplicons were pooled in equimolar amounts and paired-end sequenced on an Illumina PE300/PE250 platform (Illumina, San Diego, CA, USA) according to the standard protocols of Majorbio Bio-Pharm Technology Co., Ltd. (Shanghai, China).

### 2.6. Sequencing Data Processing and Analysis

The quality control and emergence of double-ended reads were achieved by Fastp [12] and FLASH [13] according to the overlapping relationship between the double-ended reads, after which, high-quality sequences were de-noised by DADA2 [14] to obtain amplicon sequence variants (ASVs). To minimize the effects of sequencing depth on the alpha and beta diversity measures, the number of sequences from each sample was rarefied to 20,000, which still yielded an average Good’s coverage of 97%. The taxonomic assignment of the ASVs was conducted in accordance with the naive Bayes consensus taxonomy classifier implemented in Qiime2 and the SILVA 16S rRNA database (v138) [15].

### 2.7. Statistical Analysis

The intergroup discrepancies of PRBWC; production performance parameters including BW_0_ and BW_21_, BCS_0_, and BCS_21_; average daily milk yield during the first 21 (ADMY_21_) and 147 (ADMY_147_) days in milk; and all 19 abovementioned serum indicators were determined by independent-sample *t*-tests with SPSS 26.0 (IBM, Armonk, NY, USA). The correlation matrix among PRBWC, significant production parameter BSC_21_, and serum biomarkers including GLU, ALB, and LBP was determined by Pearson’s correlation analysis and plotted by GraphPad Prism 9.0.2 (Boston, MA, USA). A *p*-value less than 0.05 was deemed to be statistically significant.

Alpha diversity indices, including Shannon, Chao1, and ACE, were calculated by Mothur v1.30.1 [16], and the intergroup comparison was performed by the Wilcoxon rank-sum test. Beta diversity was demonstrated by principal coordinate analysis (PCoA) based on the Bray–Curtis distances using the Vegan v2.5-3 package, using analysis of similarities (ANOSIM) to determine intergroup discrepancies. Both alpha diversity and beta diversity analyses were performed on ruminal fluid samples on Day 0 (RF0), ruminal fluid samples on Day 21 (RF21), fecal samples on Day 0 (FE0), and fecal samples on Day 21 (FE21).

Additionally, the following taxonomic analyses were merely performed on FE0. Wilcoxon’s rank-sum test was used to determine differential bacterial genera between the groups. The linear discriminant analysis effect size (LEfSe, LDA > 3.5) enabled the identification of significantly abundant taxa (phylum to genera) of bacteria between groups. The Pearson correlation analysis was used to assess the interactions between bacterial genera and intergroup differential indicators for 42 cows enrolled in the taxonomic analysis. The co-occurrence networks were constructed to explore the internal community relationships across the samples [17]. A correlation between two nodes was statistically robust if Spearman’s correlation coefficient was over 0.6 or less than −0.6 and the *p*-value was less than 0.05. The microbiome function was subsequently predicted with PICRUSt 2 [18]. Intergroup significantly enriched pathways were eventually identified by Wilcoxon’s rank-sum test based on the Kyoto Encyclopedia of Genes and Genomes (KEGG), MetaCyc Metabolic Pathway (METACYC), and Clusters of Orthologous Genes (COGs) databases.

## 3. Results

The PRBWC and the parameters used for its calculation of every experimental cow are listed in Table S3. The PRBWC values of all 59 experimental cows ranged from −20.46% to 1.10%. The mean PRBWC of 21 cows in the H-PRBWC group was −3.05 ± 0.43%, while it was −14.18 ± 0.81% in the L-PRBWC group.

### 3.1. Production Performance

As shown in Table 1, neither ADMY_21_ nor ADMY_147_ showed statistically significant intergroup discrepancies. However, both the BW_21_ and BCS_21_ of the H-PRBWC group were significantly higher than that of the L-PRBWC group (BW_21_, *p* = 0.02; BCS_21_, *p* < 0.01), while neither BW_0_ or BCS_0_ demonstrated statistically significant intergroup discrepancies.

### 3.2. Serum Biomarkers

The comparative results of serum biomarkers between groups on Day 21 are presented in Table 2. The GLU concentration of the H-PRBWC group was significantly higher (*p* = 0.05). The ALB level was significantly higher in the H-PRBWC group (*p* < 0.01), while the concentration of LBP was significantly lower in the H-PRBWC group (*p* = 0.03).

### 3.3. Correlation Matrix of PRBWC and Intergroup Differential Indicators on Day 21

**Figure 1:**
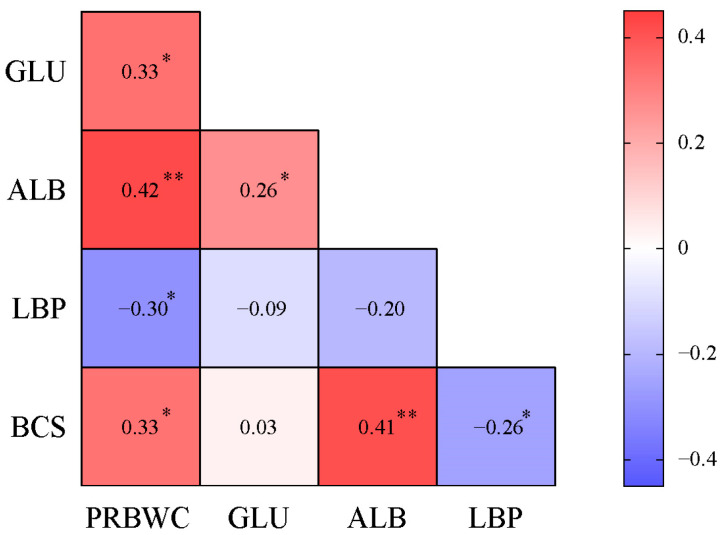
Correlation matrix of PRBWC and the corresponding intergroup significantly differential parameters on Day 21. **, *p* < 0.01; *, 0.01 ≤ *p* < 0.05. Values in the cell are Pearson’s correlation coefficients. PRBWC, postpartal relative body weight change; GLU, serum glucose on Day 21; ALB, serum albumin on Day 21; LBP, serum lipopolysaccharide-binding protein on Day 21; BCS, body condition score on Day 21.

Production parameters and serum biomarkers exhibiting significant intergroup discrepancies on Day 21 were identified as intergroup differential indicators. Figure 1 demonstrates the correlation matrix of the PRBWC and intergroup differential indicators observed on Day 21. PRBWC showed a positive association with BCS (*r* = 0.33, *p* = 0.01), GLU (*r* = 0.33, *p* = 0.01), and ALB (*r* = 0.42, *p* < 0.01), while demonstrating a negative association with LBP (*r* = −0.30, *p* = 0.02). Furthermore, BCS was also positively correlated with ALB (*r* = 0.41, *p* < 0.01) and had a negative correlation with LBP (*r* = −0.26, *p* = 0.05). Additionally, GLU exhibited a positive correlation with ALB (*r* = 0.26, *p* = 0.05).

### 3.4. Intergroup Diversity Analyses at Diverse Gastrointestinal Sites on Different Days

**Figure 2:**
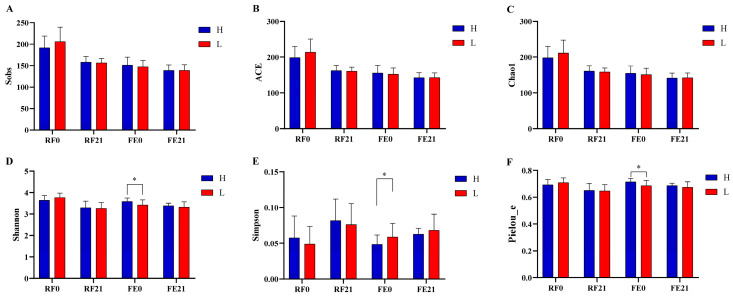
Discrepancies in bacterial alpha diversity indices between the H-PRBWC group and the L-PRBWC group. (**A**) Sobs, (**B**) ACE, (**C**) Chao1, (**D**) Shannon, (**E**) Simpson, and (**F**) Pielou_e. *, 0.01 ≤ *p* < 0.05. RF0, ruminal fluid samples on Day 0; RF21, ruminal fluid samples on Day 21; FE0, fecal samples on Day 0; FE21, fecal samples on Day 21. H, H-PRBWC group; L, L-PRBWC group.

As shown in Figure 2, alpha diversity indices, including Sobs, ACE, Chao1, Shannon, Simpson, and Pielou_e, were employed to assess the intergroup discrepancy in alpha diversity between the H- and L-PRBWC groups regarding both timepoints (Day 0 or Day 21) and gastrointestinal sites (rumen or rectum). No significant intergroup differences in alpha diversity were observed across the RF0, RF21, and FE21 comparisons (Table S4). However, the H-PRBWC group exhibited significantly higher Shannon and Pielou_e and lower Simpson indices of the bacterial communities only in FE0 (Figure 2D–F).

**Figure 3:**
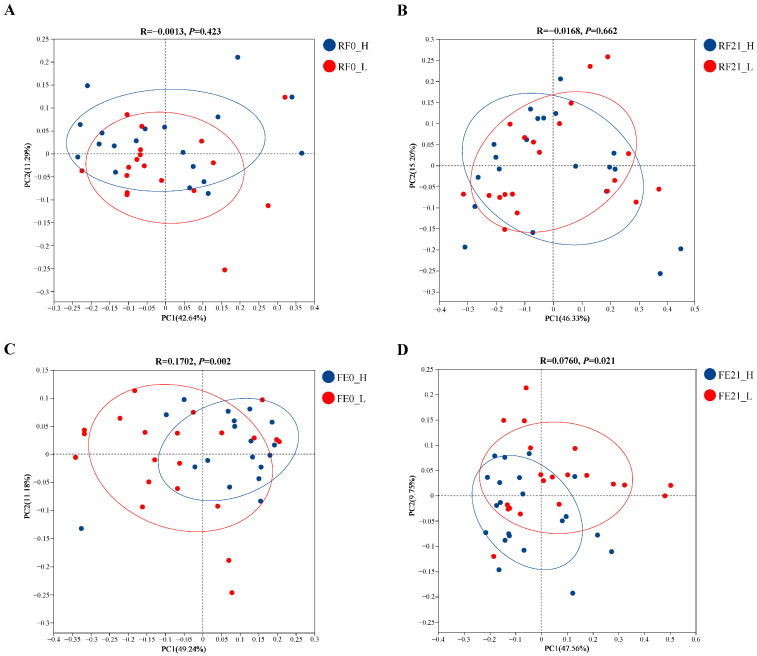
Cluster analysis of rumen or fecal bacteria between groups. (**A**) RF0_H, ruminal fluid samples of the H-PRBWC group on Day 0; RF0_L, ruminal fluid samples of the L-PRBWC group on Day 0. (**B**) RF21_H, ruminal fluid samples of the H-PRBWC group on Day 21; RF21_L, ruminal fluid samples of the L-PRBWC group on Day 21. (**C**) FE0_H, fecal samples of the H-PRBWC group on Day 0; FE0_L, fecal samples of the L-PRBWC group on Day 0. (**D**) FE21_H, fecal samples of the H-PRBWC group on Day 21; FE21_L, fecal samples of the L-PRBWC group on Day 21.

The four comparisons demonstrated in Figure 3 were relatively robust, since the contribution of each comparison to their corresponding total variance in each graph was over 50% (PC1 + PC2). However, the intergroup discrepancy in either RF0 or RF21 was not significant (*p* > 0.05). Furthermore, the R value of FE0 was far larger than that of FE21, RF0, and RF21, indicating the intergroup discrepancy was more predominant than the intragroup discrepancy of FE0. Above all, the following intergroup analyses were only performed on FE0 regarding the results of both the alpha and beta diversity intergroup analyses.

### 3.5. Bacterial Richness and Structural Composition of FE0

**Figure 4:**
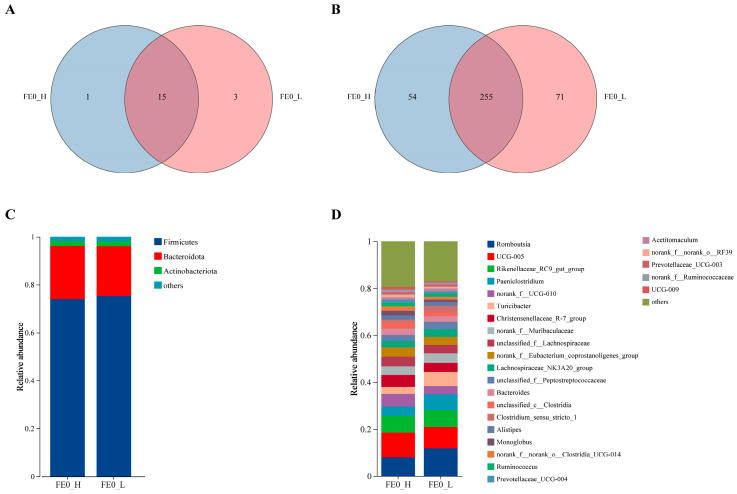
Fecal bacterial communities of the H-PRBWC group and L-PRBWC group on Day 0. Venn diagrams illustrating the unique and common bacteria between groups at the phylum (**A**) and genus (**B**) levels. The composition of dominant bacteria at the phylum (**C**) and genus (**D**) levels in both the H-PRBWC and the L-PRBWC groups. FE0_H, fecal samples of the H-PRBWC group on Day 0; FE0_L, fecal samples of the L-PRBWC group on Day 0.

In this study, a total of 380 bacterial genera (Figure 4B) spanning 19 bacterial phyla (Figure 4A) were identified in FE0. Of these, 255 genera from 15 phyla were found in both groups. Bacteria of FE0 that had a relative abundance of over 1% at either the phylum level or the genus level are demonstrated in Figure 4C,D. Both groups showed a predominant phylum level of Firmicutes (H-PRBWC, 73.99%; L-PRBWC, 75.26%), Bacteroidota (H-PRBWC, 22.21%; L-PRBWC, 20.87%), and Actinobacteriota (H-PRBWC, 1.98%; L-PRBWC, 1.94%) (Figure 4C). A total of 25 genera were predominant in both groups (Figure 4D).

### 3.6. Intergroup Differential Bacterial Genera of FE0

**Figure 5:**
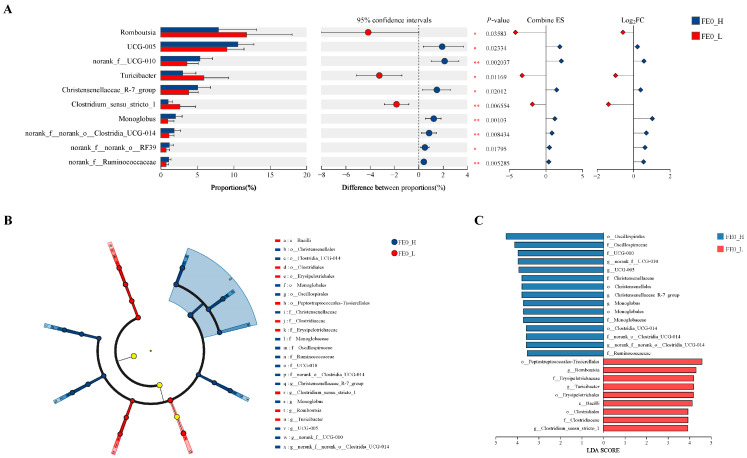
Differential analysis of fecal bacterial composition between the H-PRBWC group and the L-PRBWC group on Day 0. (**A**) Dominant genera in the H- and L-PRBWC groups identified by Wilcoxon’s rank-sum test. *, *p* < 0.05; ** *p* < 0.01. (**B**) Cladogram demonstrating the dominant bacterial branches in the H- and L-PRBWC groups (**C**) Histograms of linear discriminant analysis score based on categorical information. FE0_H, fecal samples of the H-PRBWC group on Day 0; FE0_L, fecal samples of the L-PRBWC group on Day 0.

To identify the intergroup-dominant bacteria, the Wilcoxon rank-sum test and LEfSe were utilized. As illustrated in Figure 5A, the top 10 intergroup significantly different genera (*p* < 0.05) were ranked by their relative abundances, among which *UCG-005*, *norank_f__UCG-010*, *Christensenellaceae_R-7_group*, *Monoglobus*, *norank_f__norank_o__Clostridia_UCG-014*, *norank_f__norank_o__RF39*, and *norank_f__Ruminococcaceae* were found to be more abundant in the H-PRBWC group, while *Romboutsia*, *Turicibacter*, and *Clostridium_sensu_stricto_1* were more abundant in the L-PRBWC group. With the criteria LDA > 3.5 and *p* < 0.05, 15 branches in the H-PRBWC group and 9 branches in the L-PRBWC group were identified (Figure 5B), which included five genera from the H-PRBWC group and three genera from the L-PRBWC group (Figure 5C). The intergroup differential bacteria of FE0 were determined by both Wilcoxon’s rank-sum test and LEfSe, including eight genera: *Romboutsia*, *norank_f__norank_o__Clostridia_UCG-014*, *Monoglobus*, *Clostridium_sensu_stricto_1*, *Christensenellaceae_R-7_group*, *Turicibacter*, *norank_f__UCG-010*, and *UCG-005*. The eight abovementioned genera were all predominant in both groups (Figure 4D).

### 3.7. Correlations Between FE0 Bacterial Genera and Intergroup Differential Indicators on Day 21

**Figure 6:**
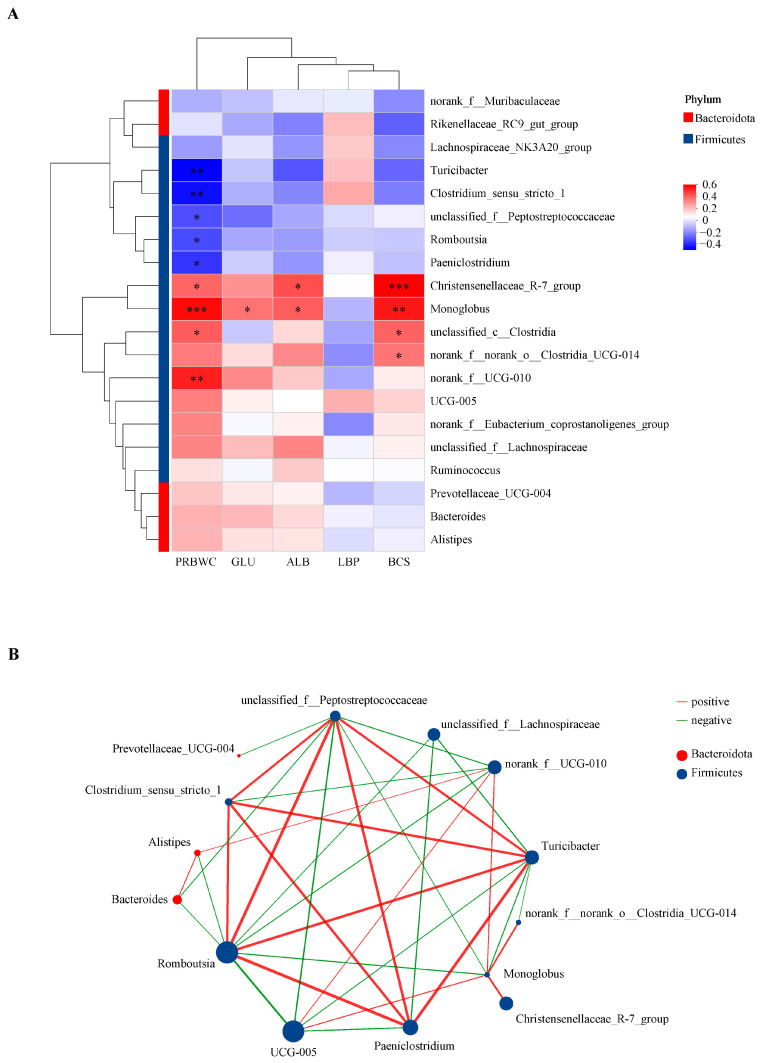
Correlation analyses within bacteria or with host-differential phenotypic traits. Genera labeled with blue color were dominant in the FE0 of the H-PRBWC group; genera labeled with red color were dominant in the FE0 of the L-PRBWC group. (**A**) The heatmap shows partial Spearman correlation coefficients between FE0 genera with the top 20 relative abundance and five host-differential phenotypic traits on Day 21. *, *p* < 0.05; ** *p* < 0.01; ***, *p* < 0.001. GLU, glucose; ALB, albumin; LBP, lipopolysaccharide-binding protein; BCS, body condition score. (**B**) Co-occurrence networks of the active microbial genera with the top 20 relative abundance, a Spearman’s coefficient > |0.6|, and a *p*-value < 0.05 between ASVs.

Intergroup differential production indicators (Table 1) and serum biomarkers (Table 2) have been detailed in previous sections. As illustrated in Figure 6A, PRBWC displayed a positive correlation with *Christensenellaceae_R-7_group*, *Monoglobus*, *unclassified_c__Clostridia*, *norank_f__UCG-01*, while exhibiting a negative correlation with *Turicibacter*, *Clostridium_sensu_stricto_1*, *unclassified_f__Peptostreptococcaceae*, *Romboutsia*, and *Paeniclostridium*. Additionally, GLU was positively correlated with *Monoglobus*, ALB showed positive correlations with both *Christensenellaceae_R-7_group* and *Monoglobus*, and BCS was positively correlated with *Christensenellaceae_R-7_group* and *Monoglobus*, *unclassified_c__Clostridia*, *norank_f__norank_o__Clostridia_UCG-014*. Among all eight intergroup differential genera, only *norank_f__norank_o__Clostridia_UCG-014* and *UCG-005* displayed no significant correlation with PRBWC. However, *Christensenellaceae_R-7_group* and *Monoglobus* displayed the most significant correlations with intergroup differential indicators.

The active bacterial network comprised 14 nodes (bacterial genera) and 35 edges (significant connections) at the genus level, filtered by a Spearman’s coefficient greater than|0.6|, a significance level of *p* < 0.05, and a relative abundance ranked in the top 20 among all genera (Figure 6B). Notably, the genus *Romboutsia* had the highest number of connections at 10, followed by *unclassified_f__Peptostreptococcaceae* with 9, *Turicibacter* with 8, and *Monoglobus* with 7. In addition, *Romboutsia*, *Turicibacter*, and *Monoglobus* were more connective and important compared with the other six intergroup differential genera (Table S5).

### 3.8. Functional Profiling Toward Bacteria of FE0

**Figure 7:**
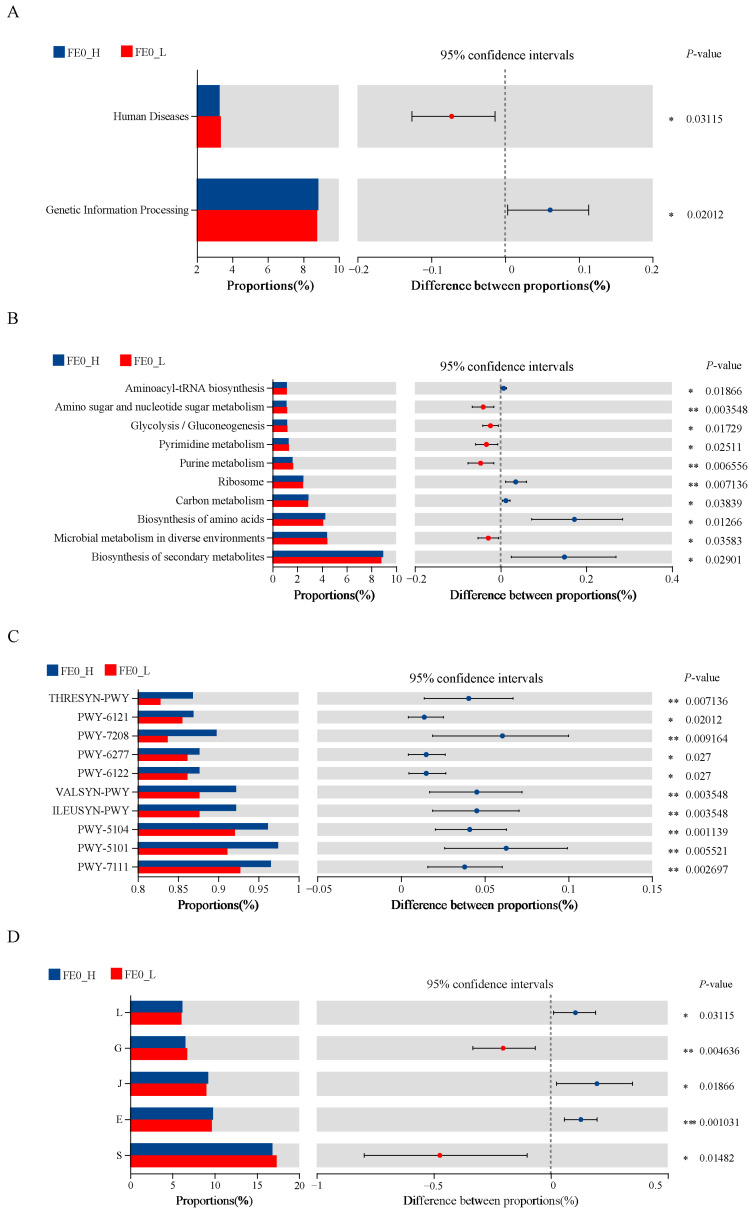
Differential analysis of fecal bacterial functions between the H-PRBWC group and the L-PRBWC group on Day 0. Differential functions are annotated in the pathways of (**A**) KEGG Level 1, (**B**) KEGG Level 3, (**C**) METACYC, and (**D**) COGs. FE0_H, fecal samples of the H-PRBWC group on Day 0; FE0_L, fecal samples of the L-PRBWC group on Day 0. *, *p* < 0.05; ** *p* < 0.01; ***, *p* < 0.001.

To investigate the functional differences between fecal bacteria from the H-PRBWC group and the L-PRBWC group on Day 0, differential functions between groups were compared by utilizing three databases: KGEE, METACYC, and COG. Differential intergroup functions were determined by the Wilcoxon rank-sum test (*p* < 0.05). Enriched pathways ranked in the top 10 are depicted in Figure 7.

The genetic information processing pathway was significantly upregulated in the H-PRBWC group, and the human diseases pathway was significantly upregulated in the L-PRBWC group (Figure 7A) at KEGG Level 1.

As for results blasted in the pathway of KEGG Level 3, the pathways of biosynthesis of secondary metabolites, biosynthesis of amino acids, carbon metabolism, ribosome, and aminoacyl-tRNA biosynthesis were significantly upregulated in the H-PRBWC group, the pathways of microbial metabolism in diverse environments, purine metabolism, pyrimidine metabolism, glycolysis/gluconeogenesis, and amino sugar and nucleotide sugar metabolism were significantly upregulated in the L-PRBWC group (Figure 7B).

All significant upregulated METACYC pathways occurred in the H-PRBWC group, including PWY-7111, pyruvate fermentation to isobutanol (engineered); PWY-5101, L-isoleucine biosynthesis II; PWY-5104, L-isoleucine biosynthesis IV; ILEUSYN-PWY, L-isoleucine biosynthesis I (from threonine); VALSYN-PWY, L-valine biosynthesis; PWY-6122, 5-aminoimidazole ribonucleotide biosynthesis II; PWY-6277, superpathway of 5-aminoimidazole ribonucleotide biosynthesis; PWY-7208, superpathway of pyrimidine nucleobases salvage; PWY-6121, 5-aminoimidazole ribonucleotide biosynthesis I; and THRESYN-PWY, superpathway of L-threonine biosynthesis (Figure 7C).

The functions predicted by COGs revealed that E (amino acid transport and metabolism), J (translation, ribosomal structure, and biogenesis), and L (replication, recombination, and repair) were significantly upregulated in the H-PRBWC group; S (function unknown) and G (carbohydrate transport and metabolism) were significantly upregulated in the L-PRBWC group (Figure 7D).

## 4. Discussion

Intergroup discrepancies in both BW and BCS were not observed on Day 0, while the H-PRBWC group displayed both extremely significant higher BW and BCS on Day 21 (Table 1), possibly indicating a minor postpartal NEB and a better energy status at the end of the postpartum in cows with a relatively higher PRBWC. However, neither the milk production during postpartum nor of the whole experimental period showed a significant intergroup discrepancy in our study, possibly because EB was highly related to energy-corrected milk yield and percentage of milk fat, rather than milk yield [3].

Cows with severe NEB were inclined to demonstrate clinical serum signs of ketosis. The normal concentration of serum GLU ranges from 2.3 to 4.1 mmol/L; cows with serum GLU below 2.3 mmol/L may suffer from ketosis [19]. The means of serum GLU in both the H-PRBWC and L-PRBWC groups were in the normal range (Table 2). On the contrary, as for another serum marker used to diagnose ketosis, the means of serum NEFA of both groups in our study were far above the threshold of 0.4 mmol/L [20]. However, the direct serum biomarker for ketosis diagnosis is BHBA, with a criterion of more than 1200 μmol/L [21], and every cow enrolled in our study was in the normal range. The divergence among these abovementioned serum biomarkers related to energy metabolism may be the cow’s adaption to the increase in milk production in the past decades through an improvement in resilience to lipolysis.

Glucose serves as the primary energy source for all organisms. For lactating cows, GLU is primarily utilized for milk production or stored as body reserves, depending on the stage in the production cycle [22]. During the postpartum, body reserves are mobilized primarily through glycogenesis, since the increase in energy intake cannot cover the energy requirement for rapid milk production [23], so either lipolysis or glycogenesis is passively initiated by the GLU requirements. Hence, the higher GLU level on Day 21 may indicate a better energy status in the H-PRBWC group of cows. ALB is a prevalent protein found in serum that plays various biological roles, including the regulation of oncotic pressure in the blood and transportation of metabolites, hormones, and nutrients [24]. Research indicates that plasma concentrations of ALB significantly increase in cows that are fed a high-energy diet [25]. Likewise, cows with a higher proportion of concentrates in their diet exhibit elevated serum ALB levels [26]. These findings suggest that cows with greater energy intake may maintain a balanced energy level, positively influencing serum ALB concentrations. Furthermore, the classic NEB state during the postpartum compels cows to excessively mobilize adipose tissues to produce more GLU through glycogenesis. In addition, NEB reduces a cow’s capability to use the NEFA that can be oxidized to ketone bodies through incomplete oxidation or be esterified to the triglyceride deposited in the liver. Both excessive glycogenesis and fat deposition in the liver may impair hepatic function [27]. Many studies have confirmed the positive correlation between serum ALB levels and liver function [28,29,30]. Therefore, the significantly lower serum ALB level observed in the L-PRBWC group on Day 21 likely indicates a more pronounced energy deficit caused by an increase in GLU requirements. Acute-phase proteins are highly conserved and play a crucial role in the systemic regulation of defense mechanisms, coagulation, proteolysis, and tissue repair. One notable acute-phase protein, LBP, functions to alleviate inflammation by sequestering small molecules and modulating the innate immune response [31]. LBP is mainly synthesized in the liver, intestine, and adipose tissue. Adipocytes act as an intermediate storage site for lipopolysaccharide (LPS). Enhanced lipolysis may lead to a greater release of LPS from the adipose tissue into the bloodstream, ultimately resulting in a compensatory increase in serum LBP levels [32]. In our study, on Day 21, the significantly higher level of serum LBP in the L-PRBWC group may arise from the intensified lipolysis during the postpartum stage. Other serum biomarkers related to energy metabolism and hepatic function demonstrated no intergroup discrepancy; all serum biomarkers related to oxidative status and inflammatory response also demonstrated no intergroup discrepancy. The NEB state of L-PRBWC cows may not be sufficient to exhibit clinic signs regarding individual BHBA level, so a larger sample size with severer NEB individuals will be required in the following studies.

Fecal microbiota on Day 0 exhibited an effect on PRBWC: the intergroup discrepancies related to both alpha and beta diversities were significant on Day 0. The statistically significant higher Shannon index and lower Simpson index of FE0 microbiota from the H-PRBWC group of cows indicated a better diversity of the microbial community and a more robust microbial community with better resistance, resilience, and functional redundancy [33]. In addition, the significantly higher Pielou_e index displayed a more even distribution of the FE0 microbial community from the H-PRBWC group of cows. However, none of the microbiota of RF0, RF21, and FE21 demonstrated intergroup discrepancies both in the alpha and beta diversity analyses, so the following intergroup taxonomic analyses were conducted only on FE0.

The six intergroup differential genera of FE0 were finally determined as shown in Section 3.7, in which *Monoglobus*, *norank_f__UCG-010*, and *Christensenellaceae_R-7_group* were predominant in the H-PRBWC group, while *Clostridium_sensu_stricto_1*, *Turicibacter*, and *Romboutsia* were predominant in the L-PRBWC group. BHBA, as a typical indicator used to evaluate NEB, was negatively correlated with *Christensenellaceae* and positively correlated with *Romboutsia* [34]. Although BHBA showed no intergroup discrepancy in our study, the PRBWC demonstrated similar correlations with *Christensenellaceae* and *Romboutsia* (Figure 6A). Even though few studies focused on the relations between gastrointestinal bacteria and cows’ energy status, many studies revealed the correlation between the concentrate proportion of feed and the bacterial profile of the hindgut in ruminants. The relative abundance of *Monoglobus* was significantly lower in the feces of Tibetan sheep fed with a low concentrate diet (concentrate-to-forage ratio, 30:70) [35]. In another relative study, subacute ruminal acidosis induced by a high-concentrate diet (concentrate-to-forage ratio, 70:30) significantly reduced the relative abundance of *Christensenellaceae R-7 group*, *UCG-010-norank*, and *Monoglobus* in the colonic contents (feces) of Hu sheep. [36]. From another aspect, the relative abundance of *Clostridium*_*sensu*_*stricto*_*1* was significantly elevated in the ileal contents of goats provided with a diet composed of 90% concentrate [37], and the relative abundance of *Turicibacter* was also significantly increased in both the colonic and cecal contents [38], and in the cecal lumen and cecal mucosa [39] of goats provided high-concentrate (grain) diets. In the abovementioned studies, high-concentrate diets seemed to increase those bacteria that were differentially predominant in the L-PRBWC group while decreasing the ones that were differentially predominant in the H-PRBWC group. The hypothesis may be that excessive concentrates cannot be fully digested, resulting in microbiota changes in the hindgut. Cows in both the H-PRBWC and L-PRBWC groups were supplied with identical diets, so the possible difference in indigestion regarding concentrates may be attributed to the individuals’ digestive function.

*Romboutsia* was considered a potentially pro-inflammatory pathogenic bacterial genus [40], and the relative abundance of *Romboutsia* was inversely correlated with the abundance of functional genes related to the intestinal barrier [41]. Furthermore, the relative abundance of *Romboutsia* also positively correlated with blood energy biomarkers that represent the degree of mobilization of body reserves to some extent, including NEFAs, BHBA, triglycerides, and cholesterol during the peripartum [34], although BHBA and NEFA were both not significant in our study.

The predicted intergroup differential functions were confirmed in three databases by PICRUSt 2. The upregulated functions involved in the biosynthesis of secondary metabolites, nucleotide-related precursors and products, and amino acids (especially BCAA) were differentially dominant in the FE0 of the H-PRBWC group (Figure 7B,C). However, pathways enriched in nucleotide-related metabolism and carbohydrate transport and metabolism, including glycolysis/gluconeogenesis and amino sugar and nucleotide sugar metabolism, were significantly upregulated in the FE0 of the L-PRBWC group (Figure 7B–D). The more active function of carbohydrate metabolism in the fecal microbiota may be a result of the lower efficiency of digestion of carbohydrates in the foregut, so the decreased energy acquisition from feed forced cows to mobilize more body reserves, leading to a lower PRBWC.

## 5. Conclusions

PRBWC has the potential to be utilized within farm management systems as an energy parameter for evaluating and selecting cows. Parameters on Day 21 such as BCS, GLU, ALB, and LBP may be supportive for the idea that PRBWC serves as a viable metric for assessing a cow’s energy levels for depicting energy changes throughout the postpartum on an individual basis. In addition, fecal microbiota on Day 0 appears to play a role in relation to PRBWC. In the H-PRBWC group, the differentially predominant genera included *Monoglobus*, *norank_f__UCG-010*, and *Christensenellaceae_R-7_group*, whereas the L-PRBWC group displayed differentially predominant genera such as *Clostridium_sensu_stricto_1*, *Turicibacter*, and *Romboutsia*. The FE0 microbiota of cows with lower PRBWC seemed to be enriched in carbohydrate metabolism functions, which may indicate enhanced fermentation of carbohydrates in the hindgut, potentially resulting from indigestion in the foregut.

## Figures and Tables

**Table 1:** The intergroup discrepancies of production performance.

Item ^1^	Group ^2^		SEM	*p*-Value
	H	L		
BW_0_ (kg)	712.0	735.5	10.60	0.27
BW_21_ (kg)	689.9	631.1	10.76	<0.01
BCS_0_ (point)	3.32	3.18	0.04	0.09
BCS_21_ (point)	3.18	2.98	0.04	<0.01
ADMY_21_ (kg/d)	34.82	34.97	0.91	0.93
ADMY_147_ (kg/d)	40.31	43.35	0.94	0.11

^1^ BW_0_, body weight on Day 0; BW_21_, body weight on Day 21; BCS_0_, body condition score on Day 0; BCS_21_, body condition score on Day 21; ADMY_21_, average daily milk yield from Day 0 to Day 21; ADMY_147_, average daily milk yield from Day 0 to Day 147. ^2^ H, H-PRBWC group; L, L-PRBWC group.

**Table 2:** Effect of PRBWC on cow serum biomarkers.

Item ^1^	Group ^2^		SEM	*p*-Value
	H	L		
BHBA (μmol/L)	607.9	651.8	19.03	0.25
BUN (mmol/L)	5.05	4.51	0.15	0.07
GLU (mmol/L)	3.29	2.53	0.19	0.05
IGF-1 (pg/mL)	150.8	156.1	2.81	0.35
NEFA (mmol/L)	0.93	1.02	0.03	0.18
TC (mmol/L)	3.83	3.53	0.13	0.23
ALB (g/L)	33.95	32.07	0.34	<0.01
HPT (μg/mL)	312.8	335.8	7.28	0.11
TBIL (umol/L)	3.41	4.02	0.15	0.45
TP (g/L)	73.74	75.31	0.56	0.17
GSH-Px (U/mL)	189.6	185.7	6.18	0.76
MDA (nmol/mL)	2.99	2.72	0.15	0.38
SOD (U/mL)	158.4	165.3	2.31	0.14
T-AOC (mmol/L)	0.26	0.27	0.01	0.63
IL-1β (pg/mL)	32.42	44.7	4.46	0.17
IL-6 (pg/mL)	94.64	69.21	10.87	0.25
LBP (μg/mL)	126.2	157	7.12	0.03
SAA (μg/mL)	208.2	208.8	1.79	0.87
TNF-α (pg/mL)	136.8	89.55	14.50	0.1

^1^ BHBA, beta-hydroxybutyric acid; BUN, blood urea nitrogen; GLU, glucose; IGF-1, insulin-like growth factor-1; NEFA, non-esterified fatty acid; TC, total cholesterol; ALB, albumin; HPT, heparin-binding protein; TBIL, total bilirubin; TP, total protein; GSH-Px, glutathione peroxidase; MDA, malondialdehyde; SOD, superoxide dismutase; T-AOC, total antioxidant capacity; IL-1β, interleukin-1β; IL-6, interleukin-6; LBP, lipopolysaccharide-binding protein; SAA, serum amyloid A; TNF-α, tumor necrosis factor-α. ^2^ H, H-PRBWC group; L, L-PRBWC group.

## Data Availability

The data that support the findings of this study are available from the corresponding author upon reasonable request. The sequencing data are available in the NCBI under BioProject number PRJNA1248168.

## Supplementary Materials

The following supporting information can be downloaded at: https://www.mdpi.com/article/10.3390/ani15091252/s1, Table S1: Composition and nutritional levels of the diet (DM%); Table S2: Information related to serum biomarker measurements; Table S3: Results and data used for individual PRBWC calculation; Table S4: *p*-values of intergroup comparisons toward alpha diversity indices; Table S5: Detailed attributes of intergroup differential genera.
