# Supplementary material for: Effects of Postpartal Relative Body Weight Change on Production Performance, Serum Biomarkers, and Fecal Microbiota in Multiparous Holstein Cows
Source: Animals (Basel). 2025 Apr 29;15(9):1252. doi: 10.3390/ani15091252 (PMC12071173; doi:10.3390/ani15091252)
Supplement: Supplementary file 1 [file animals-15-01252-s001.zip › animals-3552841-supplementary.pdf]

animals

MDPI

Table S1. Composition and nutritional levels of the diet (DM%).

Item	Diet
Ingredient, % of DM	
Corn silage	53.13
Corn grain	4.69
Oat hay	2.18
Alfalfa hay	6.25
Soybean meal	5
Cottonseed meal	3.13
Sugar Beet pulp	2.5
Steam-flaked corn	5.63
Rumen protected soybean meal	1.88
Rumen protected nicotinic acid	0.07
Limestone	0.32
Molasses	3.13
Distillers dried grains	9.37
Methionine	0.07
Fat powder	0.47
Fat acids calcium	0.32
Buffer	0.32
Mineral and vitamin premix [1]	1.56
Nutrient, % of DM [2]	
DM, as-fed basis	46.1
CP	17
EE	4.27
Starch	25.95
Ash	8.49
NDF	37.78
ADF	17.14
NFC	37.53

[1] BW, premix provided the following per kilogram of diet: 480 mg/kg Cu, 28 mg/kg I, 1,600 mg/kg Mn, 1,800 mg/kg Zn, 13 mg/kg Se, 28 mg/kg Co, 210,000 IU/kg vitamin A, 70,000 IU/kg vitamin D, and 5,600 mg/kg vitamin E. [2] DM, dry matter; CP, crude protein; NDF, neutral detergent fiber; ADF, acid detergent fiber; EE, ether extract.

Table S2. Information related to serum biomarker measurements.

Biomarker [1]	Category	Instrument / kit	Manufacture [2]
BHBA	Energy metabolism	Hitachi 7600	Company Ⅰ
BUN	Energy metabolism	Hitachi 7600	Company Ⅰ
GLU	Energy metabolism	Hitachi 7600	Company Ⅰ
IGF-1	Energy metabolism	H041-1-1	Company Ⅱ
NEFA	Energy metabolism	A042-2-1	Company Ⅲ
TC	Energy metabolism	Hitachi 7600	Company Ⅰ
ALB	Hepatic function	Hitachi 7600	Company Ⅰ
HPT	Hepatic function	H136	Company Ⅲ
TBIL	Hepatic function	Hitachi 7600	Company Ⅰ
TP	Hepatic function	Hitachi 7600	Company Ⅰ
GSH-Px	Oxidative status	A005-1	Company Ⅲ
MDA	Oxidative status	A003-1	Company Ⅲ
SOD	Oxidative status	A001-3	Company Ⅲ
T-AOC	Oxidative status	A015-2-1	Company Ⅲ
IL-1β	Inflammatory response	KJEIA0001D	Company Ⅱ
IL-6	Inflammatory response	KJEIA0006D	Company Ⅱ
LBP	Inflammatory response	H253	Company Ⅲ
SAA	Inflammatory response	H134	Company Ⅲ
TNF-α	Inflammatory response	KJEIA0018D	Company Ⅱ

[1] BHBA, beta-hydroxybutyric acid; BUN, blood urea nitrogen; GLU, glucose; IGF-1, insulin-like growth factor-1; NEFA, non-esterified fatty acid; TC, total cholesterol; ALB, albumin; HPT, heparin binding protein; TBIL, total bilirubin; TP, total protein; GSH-Px, glutathione peroxidase; MDA, malondialdehyde; SOD, superoxide dismutase; T-AOC, total antioxidant capacity; IL-1β, interleukin-1β; IL-6, interleukin-6; LBP, lipopolysaccharide binding protein; SAA, serum amyloid A; TNF-α, tumor necrosis factor-α. [2] Company Ⅰ,Hitachi High-Technologies Corporation, Tokyo, Japan; Company Ⅱ, Beijing Kangjia Hongyuan Biotechnology Co., Ltd., Beijing, China; Company Ⅲ, Jiancheng Bioengineering Institute, Nanjing, China.

Table S3. Results and data used for individual PRBWC calculation [1].

Cow	Group	BW_0 (kg)	BW_{21} (kg)	PABWC (kg)	PRBWC (%)
1	H	638	645	7.0	1.10
2	H	790.5	796	5.5	0.70
3	H	662	656	-6.0	-0.91
4	H	652	643	-9.0	-1.38
5	H	616	606	-10.0	-1.62
6	H	715	700	-15.0	-2.10
7	H	656	642	-14.0	-2.13
8	H	705	689	-16.0	-2.27
9	H	795	775	-20.0	-2.52
10	H	698.5	680	-18.5	-2.65
11	H	702	682	-20.0	-2.85
12	H	756.5	732	-24.5	-3.24
13	H	672	646	-26.0	-3.87
14	H	650	623	-27.0	-4.15
15	H	778	745	-33.0	-4.24
16	H	667	635	-32.0	-4.80
17	H	674	640	-34.0	-5.04
18	H	763	723	-40.0	-5.24
19	H	928	876	-52.0	-5.60
20	H	784	740	-44.0	-5.61
21	H	649.5	613	-36.5	-5.62
22	/	693	654	-39.0	-5.63
23	/	785	740	-45.0	-5.73
24	/	713	670	-43.0	-6.03
25	/	712	669	-43.0	-6.04
26	/	797.5	748	-49.5	-6.21
27	/	705	660	-45.0	-6.38
28	/	770	717	-53.0	-6.88
29	/	634.5	590	-44.5	-7.01
30	/	665	618	-47.0	-7.07
31	/	675	624	-51.0	-7.56
32	/	637.5	587	-50.5	-7.92
33	/	873	798	-75.0	-8.59
34	/	692.5	631	-61.5	-8.88
35	/	796	724	-72.0	-9.05
36	/	694	631	-63.0	-9.08
37	/	798.5	722	-76.5	-9.58
38	/	743.5	672	-71.5	-9.62
39	L	716	646	-70.0	-9.78

40	L	767	692	-75.0	-9.78
41	L	690	621	-69.0	-10.00
42	L	669.5	601	-68.5	-10.23
43	L	756.5	677	-79.5	-10.51
44	L	739	653	-86.0	-11.64
45	L	610	538	-72.0	-11.80
46	L	756	665	-91.0	-12.04
47	L	840	736	-104.0	-12.38
48	L	823	721	-102.0	-12.39
49	L	773	672	-101.0	-13.07
50	L	755	654	-101.0	-13.38
51	L	755	645	-110.0	-14.57
52	L	617	526	-91.0	-14.75
53	L	740	621	-119.0	-16.08
54	L	796	655	-141.0	-17.71
55	L	736	600	-136.0	-18.48
56	L	654	533	-121.0	-18.50
57	L	810	649	-161.0	-19.88
58	L	697	555	-142.0	-20.37
59	L	745.5	593	-152.5	-20.46

[1] BW_0, body weight on day 0; BW_{21}, body weight on day 21; PABWC, postpartal absolute body weight change; PRBWC, postpartal relative body weight change. H, H-PRBWC group; L, L-PRBWC group.

Table S4. *P*-values of intergroup comparisons toward alpha diversity indices [1].

Item [2]	RF0	RF21	FE0	FE21
Ace	0.25	0.62	0.83	0.93
Chao	0.31	0.51	0.66	0.88
Sobs	0.27	0.58	0.60	0.80
Shannon	0.07	0.62	0.02	0.40
Pielou_e	0.13	0.76	0.02	0.49
Simpson	0.25	0.66	0.03	0.58

[1] Numbers in cells represents corresponding *P*-values. [2] Alpha diversity indices. RF0, ruminal fluid samples on day 0; RF21, ruminal fluid samples on day 21; FE0, fecal samples on day 0; FE21, fecal samples on day 21.

Table S5. Detailed attributes of intergroup differential genera [1].

Genera	RB (%)	Degree	Clustering	C-D	C-C	C-B
Romboutsia	10.00	10	0.47	0.77	0.81	0.21
UCG-005	9.77	6	0.80	0.46	0.65	0.01
Turicibacter	4.60	8	0.54	0.62	0.72	0.12
UCG-010	4.43	6	0.60	0.46	0.65	0.05
Christensenellaceae_R-7_group	4.42	1	0.00	0.08	0.42	0.00
Clostridium_sensu_stricto_1	1.91	5	0.80	0.38	0.59	0.01
Monoglobus	1.49	7	0.48	0.54	0.68	0.21
Clostridia_UCG-014	1.47	2	1.00	0.15	0.48	0.00

[1] RB, relative abundance; Degree, the number of connections with the specific genus; Clustering, clustering index to evaluate connection condition with adjacent genera; C-D, degree centrality; C-C, closeness centrality; C-B, betweenness centrality. Centrality was to evaluate the importance of the specific genus in network analysis.

Disclaimer/Publisher's Note: The statements, opinions and data contained in all publications are solely those of the individual author(s) and contributor(s) and not of MDPI and/or the editor(s). MDPI and/or the editor(s) disclaim responsibility for any injury to people or property resulting from any ideas, methods, instructions or products referred to in the content.
